# Surgery

**Published:** 1866-04

**Authors:** A. C. Post


### SELECTED ARTICLES.

### SURGERY.

*The Gutta-Percha Shoe in the Treatment of Talipes.* By Prof. A. C. Post, M. D.

Aout sixteen years ago I was treating a little girl for talipes varus, with a modification of Scarpa's shoes, which I was then in the habit of employing, when troublesome ulceration of the integument occurred from the pressure of the straps which were used to secure the shoes upon the feet. It was evidently a matter of necessity to omit for a time the use of the shoes, until the ulcerated surfaces should have an opportunity to heal. I was much chagrined by the prospects of a long delay in the treatment, especially as the patient resided in the country, and it was quite inconvenient to the parents to keep her for a long time in the city. I was led to reflect on the best means of preventing a return of the deformity towards its original condition, during the period when I should be obliged to suspend the use of Scarpa's shoes. It occurred to me, that a splint or shoe of gutta-percha might be applied in such a manner as to maintain the improvement which had already been gained by the treatment, if not to make some further advance towards a cure of the deformity. I accordingly contrived and applied such an instrument, keeping it in place by means of a roller bandage. I found that by this means the feet could be maintained in a good position, with very little inconvenience to the little patient; and under appropriate dressings, the ulcerated surfaces soon healed. To my surprise, the deformity yielded more readily to the new treatment than it had done while Scarpa's shoes had been worn, and I felt no disposition to return to the use of the spring shoes after the ulcers had healed. From my experience of the benefits of the simple contrivance which I had used in the case just alluded to, I was induced to employ it in similar cases which were presented to me; and the results were so entirely satisfactory, that I have ever since employed shoes or splints of similar construction in the treatment of infantile club-foot, in preference to the spring shoes which surgeons ordinarily employ for the same purpose. The material which I ordinarily use in the construction of these shoes, is a gutta-percha sheet from a sixteenth to an eighth of an inch in thickness. It is cut of such a shape as to adapt itself to the sole and sides of the foot, leaving a space uncovered on the dorsum of the foot equal to about one-third of the breadth of the foot; it is also adapted to the sides of the leg, extending up two-thirds of the distance to the knee, and leaving a narrow space uncovered before and behind, each space so uncovered being about one-sixth of the circumference of the leg. The material is readily moulded to the shape of the limb, by immersing it for a few seconds in water, at a temperature of 100° Fahrenheit. I am in the habit of moulding the shoes thus heated, over a wooden last made for the purpose. The last is not made after the fashion of a bootmaker's last, but it is shaped like the natural leg and foot, except that the outer side of the foot is made to correspond with the inner, thus obviating the necessity of having separate lasts for the right and left foot. I have sometimes used similar shoes made of felt stiffened with shellac, as manufactured by Dr. Ahl, of Southern Pennsylvania. In order to mould the felt, it must be dipped in water at nearly a boiling temperature, and the hands require to be protected by means of cotton gloves wet with cold water. I am rather inclined to prefer the gutta-percha shoes to those which are made of felt, especially as the former material is more conveniently moulded to its proper shape.

I generally commence the treatment of infantile club-foot by the subcutaneous division of the tendo-Achillis, after which I apply a strip of isinglass plaster over the small wound of the skin. I then have the foot held by an assistant as nearly as possible in its normal position, and while it is so held, I carefully apply a roller bandage, so as to cover the foot and leg, beginning the application on the outer side of the ankle. I then apply the gutta-percha shoe, an assistant grasping the leg with one hand, pressing the upper part of the shoe against the sides of the limb, and with the other hand pressing the sole of the shoe against the sole of the foot. While the shoe is then firmly pressed against the leg and foot, I apply a roller bandage firmly, so as to secure it in its place. After the lapse of twenty-four to forty-eight hours, I take off the bandages and shoe, wash the foot, wipe it dry, use passive motion freely in different directions, and then reapply the apparatus as before. The application is repeated at intervals of two or three days, until the foot is brought to its proper shape, when it is put up in a laced boot, lacing to the toes, and having a firm sole and stiff sides, provided with iron braces which extend nearly as high as the knee, and secured by a strap and buckle around the upper part of the leg.

The following are, in my estimation, the advantages of the gutta-percha shoe over Scarpa's shoe, and its various modifications:

1st. Its greater simplicity, and the ease with which it is made. When the material is at hand, the shoe can readily be made in fifteen minutes.

2d. It is much cheaper than the spring shoe.

3d. It is more comfortable to the patient, being lighter, exerting a less injurious pressure, and being less likely to be kicked off a restless child.

4th. It is much less likely to occasion excoriation or ulceration of the integuments.

5th. It expedites the cure, giving a better support to the foot, and bringing it more readily into its normal position.—*N. Y. Medical Record.*

